# Literacy and stigma of suicide in Bangladesh: Scales validation and status assessment among university students

**DOI:** 10.1002/brb3.2432

**Published:** 2021-12-01

**Authors:** S. M. Yasir Arafat, Fahad Hussain, Md. Faruk Hossain, Md. Aminul Islam, Vikas Menon

**Affiliations:** ^1^ Department of Psychiatry Enam Medical College and Hospital Dhaka Bangladesh; ^2^ Department of Pharmacy Noakhali Science and Technology University Noakhali Bangladesh; ^3^ Department of Media Studies and Journalism University of Liberal Arts Bangladesh (ULAB) Dhaka Bangladesh; ^4^ Department of Psychiatry Jawaharlal Institute of Postgraduate Medical Education and Research (JIPMER) Puducherry India

**Keywords:** psychometric properties, reliability, stigma, suicide in Bangladesh, suicide literacy, validity

## Abstract

**Background:**

Increased suicide literacy and reduced stigma toward suicide facilitate the care‐seeking for suicidal behavior. However, no attempt has been identified to determine these two vital aspects in Bangladesh.

**Objectives:**

We aimed to validate the literacy of suicide scale and stigma of suicide scale into Bangla along with the determination of the level of suicide literacy and stigma toward suicide.

**Methods:**

We conducted this study between April and June 2021. Data were collected from medical school and university students by *Google form*. We used a questionnaire consisting of four segments (i.e., sociodemographic questionnaire, a questionnaire for suicidal behavior, Bangla literacy of suicide scale [LOSS‐B], and Bangla stigma of suicide scale [SOSS‐B]) for data collection. We tested the psychometric properties of the scales in a sample of 529 students and examined factors associated with suicide stigma and literacy.

**Results:**

The mean age of the students was 22.61 ± 1.68 (range 18–27) years, 274 (51.8%) were males, 476 (89.9%) were graduate students, and 490 (92.6%) were unmarried. The mean score of LOSS was 4.27 ± 1.99 ranging from 0 to 10. Factor analysis revealed acceptable psychometric properties of SOSS‐B. The literacy was significantly higher in females, students of medicine, having a family history of suicidal attempts, and a history of student nonfatal attempts, while stigma was significantly lower among the females and a history of past attempts.

**Conclusions:**

This study revealed the level of literacy and stigma and culturally tested the psychometric properties of the LOSS‐B and SOSS‐B among university students in Bangladesh.

## Abstract 2

This is the first attempt to determine the level of suicide literacy and stigma in Bangladesh. We tested the psychometric properties of the literacy and stigma of suicide scales in Bangla. The literacy was significantly higher in females, students of medicine, having a family history of suicidal attempts, and a history of student non‐fatal attempts.

## 1 INTRODUCTION

Suicide is an important cause of mortality across the globe. More than 8,00,000 people are dying every year by suicide, and it is the second leading cause of death among the 15–29 years old age group (World Health Organization [WHO], 2014). Suicide attempts are more than 20 times higher than the suicides that cause significant economic burdens (WHO, 2014). It happens as a result of a complex interaction between several risk factors (WHO, 2014; Zalsman et al., 2016). Studies revealed that 90% of the suicides have at least one psychiatric disorder (Zalsman et al., 2016). Therefore, psychiatric disorders have been identified as an important risk factor for suicide, and adequate psychiatric care is an important suicide prevention strategy (Zalsman et al., 2016). However, several factors such as a higher level of stigma toward suicidal behavior, inadequate suicide and/or mental health literacy, cultural representation, and criminal legal status hinder the care‐seeking for suicidal behaviors (Aldalaykeh et al., 2020; Batterham et al., 2013a, 2013b; Calear et al., 2014; United for Global Mental Health, 2021).

The literacy of suicide scale‐short form (LOSS‐SF) was developed by Calear et al. in 2012, and the stigma of suicide scale‐short form (SOSS‐SF) was developed by Batterham et al. in 2013a in Australia (Aldalaykeh et al., 2020; Batterham et al. 2013a, 2013b; Calear et al., 2012 cited in Batterham et al., 2013b). These instruments were validated and used in several languages such Arabic (Aldalaykeh et al., 2020), Chinese (Han et al., 2017), and Turkish (Oztürk et al., 2017) to assess the suicide literacy and stigma toward suicide. These instruments are short, focused, and easily administrable.

Bangladesh is a densely populated country in South Asia with more than 160 million populations (World Population Review, nd), while Bangla is the sixth most widely used language (Ethnologue, nd). The country lags far behind in formulating a national suicide prevention strategy and prioritizing suicide prevention (Arafat, 2021). Suicide has still been a criminal offense in the legal system that hinders the disclosure of suicidal behavior and help‐seeking for that. There is no suicide surveillance system, and quality suicide data are scarce in the country (Arafat, 2019a; United for Global Mental Health, 2021). There are also sporadic and scanty prevention activities and only one psychiatric setup specialized for suicide prevention (Arafat, 2019a). None of the prevention initiatives is available 24/7. The country has 0.13 psychiatrists per 100,000 population, which is much lower to cope with the mental health burden (Mental Health ATLAS, 2017). The case–control psychological autopsy study revealed that 61% of suicides had at least one psychiatric disorder; among them, only 13% were under psychiatric care and diagnosed before their death (Arafat et al., 2021). There have been strong negative cultural attitudes and stigma toward suicide resulting in the hindrance of suicidal behavior and delayed or lack of help‐seeking from the mental health professionals (Arafat, 2019a). However, there is no available instrument in Bangla to measure literacy and the stigma of suicide in the country. Against this background, we aimed to adapt and validate the literacy of suicide scale and stigma of suicide scale into Bangla. We also aimed to determine the level of suicide literacy and stigma toward suicide in Bangladesh. The study results would act as a baseline reference as well as facilitate further studies and policy‐making while formulating the national suicide prevention strategy in the country.

## 2 METHODS

### 2.1 Study place and procedure

We conducted this cross‐sectional study among the university students of Bangladesh between April and June 2021. We selected three institutions conveniently, namely Enam Medical College (EMC), Dhaka; University of Liberal Arts Bangladesh (ULAB), Dhaka; and Noakhali Science and Technology University (NSTU), Noakhali for data collection. The EMC is a private medical college located at the periphery of the capital city, Dhaka; ULAB is a private university located at the center of the capital; and NSTU is a public university located about 200 km away from the capital. This study was conducted through an online survey using *Google form*. We created the Google form mentioning the items obtained from the instrument adaptation process and forwarded the links to the students of the selected institutions. During the COVID‐19 pandemic, there were online classes in the institutions that facilitate the link forwarding as the students have regular WhatsApp/Messenger groups. We approached students conveniently and requested them to forward the link to other batch mates. Therefore, the responses included other few institutions. We mentioned a summary of the survey including study objectives and investigators’ details at the starting of the survey, followed by the consent form. Native Bangla‐speaking Bangladeshi university students aged 18 years and above were included in the study. Foreign students were excluded from the study.

Data were collected between May 23 and June 10, 2021. Data cleaning was done by the first author and cross‐checked by the second author. In the current study, the first and the second authors acted as gate‐keeper, and only they had the access to data. After cleaning, data were stored in Microsoft Excel software. The responses were collected anonymously. A total of 534 students participated in the survey. However, five students did not have consent resulting in inaccessibility of contents. Therefore, we dropped them and analyzed 529 responses. Duplications were checked by e‐mail addresses, and no duplicate responses were identified.

### 2.2 Instruments

#### 2.2.1 Sociodemographic questionnaire

It consisted of age, sex, marital status, educational attainment, institution, faculty, subject, studying year, religion, family type (nuclear/joint), family income, chronic illness, mental disorder, medication history, and exposer to suicide (family member or any familiar person).

#### 2.2.2 Questionnaire for suicidal behavior

It consisted of suicidal thoughts in the past year and life time, suicidal plan, attempt, and disclosure of suicidal thoughts.

#### 2.2.3 Bangla literacy of suicide scale (LOSS‐B)

It was adapted from the original instrument that was developed by Calear et al. in 2012 consisting of 26 items with short‐form having 12 items (Aldalaykeh et al., 2020; Batterham et al., 2013a, 2013b; Calear et al., 2012). The 12 statements have three responding options, namely yes, no, and do not know. The total score is calculated based on the correct answer. Therefore, the total score ranges from 0 to 12. It assesses literacy about suicide in four dimensions: signs and symptoms (three items), nature of suicide (four items), risk factors (three items), and preventive measures (two items) (Calear et al., 2012; Chan et al., 2014). Due to the response pattern (yes, no, and do not know), internal consistency form of reliability and factor analysis are not justified for LOSS. Our adapted LOSS‐B has true statements in 2, 4, 6, and 8 numbers, and rests are false.

#### 2.2.4 Bangla stigma of suicide scale

It was adapted from the original instrument that was developed by Batterham et al. (2013a, 2013b) and consists of 16 items. The scale contains a common descriptor, that is, “people who die by suicide are:” The respondents would mention their amount of agreement with each descriptor by indicating one from the five mentioned options, that is, strongly disagree (1), disagree (2), neutral (3), agree (4), and strongly agree (5). The scale has three subscales: stigmatization (eight items), isolation/depression (four items), and normalization/glorification (four items). The score of each subscale score is determined by calculating the mean of responses to the items within the subscale (Aldalaykeh et al., 2020). The subscales revealed an acceptable internal consistency measured by Cronbach's alpha greater than 0.78 (Batterham et al., 2013a, 2013b).

### 2.3 Adaptation of LOSS and SOSS into Bangla

The adaptation of the instruments was performed following standard recommendations forward‐backward translation (Arafat et al., 2016; Beaton et al., 2000). One medical graduate who was informed about the study and one university graduate who was disguised about the study were involved in forward translations. The forward translated versions were compared, contrasted, and a single forward translated version was compiled after addressing the discrepancies. Then, the compiled forward version was translated back into English by another medical graduate and another university graduate. All the translators are native speakers of Bangla and fluent in English. As per the recommendation, we created a complied back‐translated version following the same methods while compiling the forward translations. Subsequently, all four versions were assessed by the expert committee, which was formed for this study. The expert committee reviewed and suggested the final adaptation of the instruments. With that version, pretesting was done among the 34 persons and adaptation was finalized. During adaptation, several items were modified. We linguistically modified item numbers 2 and 4 and the scoring of item 11 on the LOSS scale. We included, in item 2 (“seeing a psychiatrist or psychologist can help prevent someone from suicide”), mental health professional as defined in the mental health act of Bangladesh instead of a psychiatrist to broaden the services settings as the country has an inadequate number of psychiatrists (Mental Health Act, 2018). We included, in item 4 (“there is a strong relationship between alcoholism and suicide”), substance addiction instead of alcoholism as studies revealed that other forms of substances are major risk factors for suicide (Arafat et al., 2021). Moreover, as a Muslim majority country, alcoholism is not a major public health problem in the country. We had to change the scoring of item 11 (“men are more likely to commit suicide than women”) due to the reverse gender pattern of suicide in Bangladesh (Arafat, 2019b; WHO, 2018).

### 2.4 Data analysis

We collected 529 responses and analyzed them by Statistical Package for the Social Science version 23.0 software and Microsoft Excel (2010). Sociodemographic and suicidal behaviors were variables expressed in frequency and percentages. The psychometric properties of LOSS‐B and Bangla stigma of suicide scale (SOSS‐B) were assessed following standard recommendations (Arafat et al., 2016). The internal consistency form of reliability was measured by Cronbach's alpha coefficient, and a cut‐off of ≥0.70 was considered acceptable. Face and content validities were assessed while performing the adaptation of the instruments (Arafat et al., 2016). We tested the construct validity by exploratory factor analysis. Factor rotation was performed to identify the factors of the construct which is preloaded in the statistical package. Items with loading value <0.5 was considered to drop from the construct as considered in previous studies (Aldalaykeh et al., 2020; Batterham et al., 2013a, 2013b). Independent *t*‐test was performed to assess the differences between the groups.

### 2.5 Ethical aspects

We conducted the study complying with the declaration of Helsinki (1964). Before starting the study, we took formal permission from the instrument developing authors (Batterham et al., 2013a, 2013b) on April 13, 2021. Ethical approval was taken from the ethical review committee of Enam Medical College on May 22, 2021 (EMC/ERC/2021/05‐1). Informed consent was obtained from the participants before entering the main questionnaire. Data were collected avoiding the identification details of the respondents.

## 3 RESULTS

The mean age of the students was 22.61 ± 1.68 years ranging from 18 to 27 years. Among the 529 university students, 274 (51.8%) were males, 476 (89.9%) were graduate students, 490 (92.6%) were unmarried, 443 (83.7%) were Muslim, and 451 (85.3%) were living in nuclear families (Table 1). The majority of the students (29.7%) were studying in the third year of graduation, followed by fourth year (16.6%), second year (16.1%), and first year (13.6%) (Table 1). The majority of the students were studying in NSTU (43.3%), followed by EMC (27.6%) and ULAB (Table 1). The mean score of LOSS was 4.27 ± 1.99 ranging from 0 to 10 where 56.7% of the students scored 0–4 and 43.3% scored above 5–10. Correct responses to items of the literacy of suicide scale are shown in Table 2. The lowest correct response was found in item 1 (1.89%), and the maximum correct response was found in item 2 (77.1%) (Table 2). The Kaiser‐Meyer‐Olkin (KMO) Measure of Sampling Adequacy was 0.83 which was statistically significant (*p* = <.0001). The internal consistency of the SOSS‐B was measured by Cronbach's alpha which was 0.76 for the stigma subscale, 0.88 for the isolation subscale, and 0.68 for the glorification subscale.

**TABLE 1:** Sociodemographic variables of respondents (*n* = 529)

Variable	Category	*n* (%)
Sex	Male	274 (51.80)
	Female	254 (48.02)
	Others	1 (0.19)
Education	HSC (grade 12)	476 (89.98)
	Graduate	53 (10.02)
Marital status	Unmarried	490 (92.63)
	Married	39 (7.37)
Religion	Islam	443 (83.74)
	Hindu	74 (13.99)
	Cristian	4 (0.76)
	Buddhist	3 (0.57)
	Others	5 (0.95)
Study year	First year	72 (13.61)
	Second year	85 (16.07)
	Third year	157 (29.68)
	Fourth year	88 (16.64)
	Fifth year	95 (17.96)
	Post graduate	32 (6.05)
Institution	NSTU	229 (43.29)
	EMC	146 (27.60)
	ULAB	102 (19.28)
	Others	52 (9.83)
Faculty	Medicine	162 (30.62)
	Science	146 (27.60)
	Social Science	108 (20.42)
	Business Studies	47 (8.88)
	Engineering	37 (6.99)
	Arts	21 (3.97)
	Educational Sciences	5 (0.95)
	Law	3 (0.57)
Family type	Nuclear	451 (85.26)
	Joint	78 (14.74)
History of mental illness	Yes	61 (11.53)
Family history of suicide attempt	Yes	51 (9.64)
Family history of suicide	Yes	30 (5.67)
History of suicidal attempt	Yes	64 (12.09)
Total		529 (100)

Abbreviations: HSC, higher secondary; NSTU, Noakhali Science and Technology University; EMC, Enam Medical College; ULAB, University of Liberal Arts Bangladesh.

**TABLE 2:** Correct responses to items of the literacy of suicide scale (*n* = 529)

Item number	Item	Dimension	*n* (%)
1	“If assessed by a psychiatrist, everyone who suicides would be diagnosed as depressed” (F)	Cause/nature	10 (1.89)
2	“Seeing a psychiatrist or psychologist can help prevent someone from suicide” (T)	Treatment/ prevention	408 (77.13)
3	“Most people who suicide are psychotic” (F)	Risk factor	130 (24.57)
4	“There is a strong relationship between alcoholism and suicide” (T)	Risk factor	334 (63.14)
5	“People who talk about suicide rarely kill themselves” (F)	Sign/ symptom	125 (23.63)
6	“People who want to attempt suicide can change their mind quickly” (T)	Sign/ symptom	176 (33.27)
7	“Talking about suicide always increases the risk of suicide” (F)	Cause/nature	97 (18.34)
8	“Not all people who attempt suicide plan their attempt in advance” (T)	Sign/ symptom	324 (61.25)
9	“People who have thoughts about suicide should not tell others about it” (F)	Treatment/ prevention	315 (59.55)
10	“Very few people have thoughts about suicide” (F)	Cause/nature	154 (29.11)
11	“Men are more likely to suicide than women” (F)	Risk factor	113 (21.36)
12	“A suicidal person will always be suicidal and entertain thoughts of suicide” (F)	Cause/nature	71 (13.42)

We performed the factor analysis by principal component analysis with varimax rotation, observed the scree plot, and fixed the instrument as three factor‐solutions. The three extracted factors covered 61.65% (35.27, 17.32, and 9.05) of variance. The factor analysis revealed three factors where item 5 (an embarrassment), 12 (pathetic), and 13 (shallow) took loadings 0.45, −0.18, and 0.15, respectively, in stigma subscale. We dropped these three items as the factor loading was <0.5. The final SOSS‐B contains 13 items (five in stigma subscale, four in isolation subscale, and four in glorification subscale). The revised item distribution into three subscales is mentioned here. Stigma subscale consists of item numbers 2, 5, 6, 12, and 13; isolation subscale consists of 4, 7, 8, and 9; and glorification subscale consists of 1, 3, 10, and 11. The factor loading is shown in Table 3. The highest agreement was noted in the isolated subscale, followed by the stigma subscale, and the lowest agreement was reported in the glorification subscale (Table 3).

**TABLE 3:** Responses and factor loading for items from the Bangla stigma of suicide scale (SOSS‐B)

					Factor loadings		
Item number	Item	Agree and strongly agree (%)	Mean	SD	Stigma	Isolation	Glorification
Q2	Cowardly	34.78	3.03	1.23	**0.74**	0.21	−0.18
Q5	Immoral	28.35	2.94	1.04	**0.58**	0.41	−0.05
Q6	Irresponsible	41.77	3.11	1.25	**0.59**	0.50	−0.15
Q12	Stupid	41.96	3.15	1.28	**0.58**	0.42	0.04
Q13	Vengeful	15.5	2.66	1.02	**0.65**	0.06	0.40
Q4	Disconnected	51.6	3.33	1.18	0.21	**0.79**	0.09
Q7	Isolated	58.2	3.45	1.16	0.24	**0.85**	0.03
Q8	Lonely	60.49	3.52	1.20	0.20	**0.88**	0.02
Q9	Lost	39.69	3.12	1.14	0.17	**0.74**	0.05
Q1	Brave	18.9	2.36	1.19	−0.41	0.23	**0.65**
Q3	Dedicated	15.12	2.57	1.03	0.15	0.23	**0.57**
Q10	Noble	3.78	1.95	0.95	−0.01	−0.23	**0.76**
Q11	Strong	6.42	2.15	1.00	−0.03	−0.01	**0.82**

*Note*: Bold values indicate factor loading >0.5.

The correlation assessment revealed that stigma and isolation subscales were positively correlated (*p* = <.001) (Table 4). The suicide literacy was significantly higher in females, students of medicine, having a family history of suicidal attempts, and a history of student nonfatal attempts, while stigma was also significantly lower among the females and a history of past attempts (Table 5). It is interesting to note a significantly higher score in the glorification subscale among the students with past attempts (Table 5).

**TABLE 4:** Correlation between Bangla literacy of suicide scale (LOSS‐B) and three subscales of Bangla stigma of suicide scale (SOSS‐B)

		LOSS	Stigma	Isolation	Glorification
LOSS	Pearson correlation		−0.09	0.08	0.03
	Significance (two‐tailed)		0.05	0.08	0.47
Stigma	Pearson correlation	−0.085		**0.61** **	−0.06
	Significance (two‐tailed)	0.050		0.00	0.19
Isolation	Pearson correlation	0.08	**0.61** **		0.09
	Significance (two‐tailed)	0.08	0.00		0.05
Glorification	Pearson correlation	0.03	−0.06	0.09	
	Significance (two‐tailed)	0.47	0.19	0.05	

*Note*: Bold values indicate positive correlation.

** Correlation is significant at the 0.01 level (two‐tailed).

**TABLE 5:** Association between demography and Bangla literacy of suicide scale (LOSS‐B) and Bangla stigma of suicide scale (SOSS‐B) score measured by independent *t*‐test

		LOSS		Stigma		Isolation		Glorification	
Variable	*n* (%)	Mean (±SD)	*p*‐Value	Mean (±SD)	*p*‐Value	Mean (±SD)	*p*‐Value	Mean (±SD)	*p*‐Value
Total		4.27 (±1.99)		14.88 (±4.19)		13.4 (±4.01)		9.03 (± 2.98)	
Sex									
Male	274 (51.80)	4 (±1.86)	**.007**	15.4 (±4.26)	**.004**	13.68 (±3.98)	.11	8.92 (±2.9)	.42
Female	254 (48.02)	4.51 (±2.11)		14.35 (±4.05)		13.13 (±4.03)		9.13 (±3.06)	
Education									
HSC (grade 12)	476 (89.98)	4.27 (±2.01)	.81	14.82 (±4.2)	.32	13.4 (±4.07)	.88	9.02 (±2.97)	.91
Graduate	53 (10.02)	4.2 (±1.88)		15.43 (±4.08)		13.49 (±3.56)		9.07 (±3.05)	
Marital status									
Unmarried	490 (92.63)	4.24 (±1.96)	.42	14.82 (±4.27)	.21	13.38 (±4.05)	.59	9.02 (±2.96)	.79
Married	39 (7.37)	4.51 (±2.45)		15.69 (±3.02)		13.74 (±3.57)		9.15 (±3.28)	
Religion									
Islam	443 (83.74)	4.23 (±1.99)	.37	14.93 (±4.25)	.61	13.42 (±4.09)	.88	9.17 (±2.68)	.63
Others	86 (16.26)	4.44 (±2.03)		14.67 (±3.91)		13.35 (±3.63)		9.01 (±3.03)	
Faculty									
Medicine	162 (30.62)	4.57 (±1.89)	**.018**	15.13 (±4.16)	.38	13.6 (±3.95)	.45	8.77 (±3.02)	.17
Others	367 (69.38)	4.13 (±2.03)		14.78 (±4.21)		13.32 (±4.05)		9.15 (±2.95)	
Family type									
Nuclear	451 (85.26)	4.3 (±2.02)	.40	14.8 (±4.17)	.27	13.43 (±4.06)	.81	9.03 (±2.91)	.92
Joint	78 (14.74)	4.09 (±1.82)		15.37 (±4.32)		13.31 (±3.75)		9.06 (±3.38)	
History of mental illness									
Yes	61 (11.53)	4.52 (±1.81)	.28	14.15 (±4.3)	.14	13.3 (±4.18)	.82	9 (±3.2)	.92
No	468 (88.47)	4.23 (±2.02)		14.99 (±4.17)		13.42 (±4)		9.04 (±2.95)	
Family history of suicide attempt									
Yes	51 (9.64)	4.82 (±1.93)	**.036**	14.08 (±3.72)	.15	13.65 (±3.57)	.66	9.69 (±3.23)	.1
No	478 (90.36)	4.21 (±2)		14.97 (±4.23)		13.38 (±4.06)		8.96 (±2.94)	
Family history of suicide									
Yes	30 (5.67)	4.5 (±1.7)	.51	15.37 (±4.04)	.52	14.43 (±3.69)	.15	10 (±3.61)	.07
No	499 (94.33)	4.25 (±2.01)		14.86 (±4.2)		13.35 (±4.03)		8.98 (±2.93)	
History of suicidal attempt									
Yes	64 (12.09)	4.81 (±2.05)	**.020**	13.91 (±3.97)	**.046**	13.02 (±4)	.40	9.8 (±2.67)	**.029**
Others	465 (87.91)	4.19 (±1.98)		15.02 (±4.21)		13.46 (±4.02)		8.93 (±3.01)	

*Note*: Bold values indicate *p* < .05.

Abbreviation: HSC, higher secondary.

## 4 DISCUSSION

### 4.1 Main findings of the study

We aimed to adapt and assess the psychometric properties of LOSS‐B and SOSS‐B and determine the level of suicide literacy and stigma toward suicide in Bangladesh. We collected data from 529 university students and tested the psychometric properties of the instruments. The mean age of the respondents was 22.61 ± 1.68, and the majority of them were male (51.8%), graduate students (89.9%), unmarried (92.6%), and Muslim (83.7%) (Table 1). The study population is quite similar to the other studies. The primary instrument development study was done among 676 Australian university populations (Batterham et al., 2013a). The Arabic validation was performed among 160 university students (Aldalaykeh et al., 2020). The Turkish validation study was done among 1100 university students (Oztürk et al., 2017). The Chinese validation study was done among 224 university students (Han et al., 2017). Among the students, only 12.1% had a suicidal attempt, 9.6% had a family history of suicide attempts, and 5.6% had a family history of suicide (Table 1). It was reported 11.3% each in the Arabic study (Aldalaykeh et al., 2020), 12.6% (attempt) and 8.5% (family history of suicidal attempt) in Turkey (Oztürk & Akin, 2018), 10.7% in Chinese college students (Li et al., 2014).

The current study revealed a low literacy in suicide as the mean value of the LOSS‐B was 4.27, and only 43.3% of the students scored more than 4 (Table 2). The students had extremely low knowledge of depression and suicidality. On the other hand, they had good knowledge regarding the role of mental health professionals and suicide prevention. The mean LOSS score and rate of passing the mean was 5.63 and 55%, respectively, in the Arabic study (Aldalaykeh et al., 2020), 5.83 and 53% in the Chinese study (Han et al., 2017), a bit lower in the Turkish study (36.9%) (Oztürkand & Akin, 2018), and higher in Australian community (>60%) (Batterhamet al., 2013a, 2013b). The existing stigma, criminality as legal status, culture, and lack of attention in educating the general population regarding suicide could be the responsible factors for this low level of literacy (Arafat et al., 2021). This low literacy is supposed to hinder the help‐seeking for suicidal behavior in Bangladesh. Universal strategies should be targeted to raise awareness and improve suicide literacy. Additionally, psychoeducation could improve the literacy status (Batterham et al., 2013a, 2013b).

The current study revealed an acceptable KMO (0.83; *p* = <.0001) as a value >0.5 has been considered as the criteria (Arafat et al., 2016). The internal consistency of the SOSS‐B was measured by Cronbach's alpha which was acceptable (>0.70) in the isolation and stigma subscale (Arafat et al., 2016). It was close to an acceptable value for the glorification subscale (0.68) (Table 3). A similar picture was revealed in the Arabic study (stigma, 0.81; isolation, 0.71; and glorification, 0.68) which could be attributed by translating words into another language that may not produce the exact meaning (Aldalaykeh et al., 2020). The Australian study (Batterham et al., 2013a), Chinese study (Han et al., 2017), and Turkish study (Oztürk et al., 2017) revealed acceptable values of internal consistencies

Due to low factor loading (<0.5), of three items (embarrassment, pathetic, and shallow) of stigma subscale we dropped these three items from the analysis, and the final SOSS‐B contains 13 items and three subscales, that is, stigma (five items; item 2, 6, 6, 15, and 16), isolation (four items; item 4, 8, 9, and 10), and glorification (four items; item 1, 3, 11, and 14). The same procedure was followed in the Chinese validation study where four items were dropped due to the poor loading in the stigma subscale and cross‐loading with the isolation subscale (Han et al., 2017). All other studies, that is, Australia (Batterham et al., 2013a, 2013b), Jordan (Aldalaykeh et al., 2020), China (Han et al., 2017), and Turkey (Oztürk et al., 2017), revealed three subscales. The observation that three items, namely embarrassment, pathetic, and shallow, had low factor loadings may indicate that either these items are not part of the same construct or that they were not clearly understood by respondents. The three factors covered 61.7% (35.3%, 17.3%, and 9.1%) of variance which was 50% (21.69%, 14.35%, and 13.96%) in Arabic validation (Aldalaykeh et al., 2020), about 60% in the primary validation study (28.5%, 18.2%, and 12.7% (Batterham et al., 2013a), and 61% (28.5%, 17.6%, and 15.9%) in another validation in Australia (Batterham et al., 2013b). The three extracted factors are similar to the other validations and cover acceptable proportions of variance.

The isolation subscale had the highest approval rate among the three subscales of SOSS‐B, followed by the stigma subscale and glorification had the lowest approval (Table 3). Broadly similar results were noted in the studies conducted in China and Turkey (Han et al., 2017; Oztürket al., 2017; Oztürk & Akin, 2018). These results suggest that students may ascribe suicide to isolation or loneliness more than providing stigmatizing or glorifying explanations. The correlation assessment revealed a similar structure to the Arabic validation (Aldalaykeh et al., 2020). The overall reliability statistics and the correlation revealed a similar structure to the Arabic validation. We postulate that this might be explained by the same religion of Jordan and Bangladesh.

Interestingly, the approval rate for all stigmatization items was higher among Bangladeshi students compared to Australian students (Batterham et al., 2013b). In contrast, the approval rates for the glorification items were similar between cultures. These findings, from a sample of university students, suggest that suicide is stigmatized among the student community in Bangladesh and that there may be a role for targeted stigma reduction efforts, similar to recommendations from the depression stigma literature (Griffiths et al., 2008). Such interventions may focus on reducing stigmatizing attitudes and increase understanding of why suicides occur. Our findings that males had lower suicide literacy while also endorsing higher stigmatizing attitudes to suicide suggest that interventions targeting suicide‐related stigma and awareness must focus on this group.

We noticed that the suicide literacy was significantly higher in females, students of medicine, having a family history of suicidal attempts, and a history of student nonfatal attempts, while stigma was also significantly lower among the females and a history of past attempts (Table 5). The results indicate that destigmatization programs or education programs for suicide prevention might have the greatest impact if they are targeted to males and to people in the wider community without direct experience of suicide. Less stigmatization in females and among the students with psychology degrees was revealed in the primary validation study (Batterham et al., 2013a). Although the current undergraduates have a negligible focus on suicide, the clinical and academic environment could be attributable to this less stigmatization among the medicine faculty students. It is noted that there was a significantly higher score in the glorification subscale among the students with past attempts (Table 5). Also, there was a similar high score in glorification among the community people of Australia with suicidal ideation; however, no change was identified in the past attempters (Batterham et al., 2013b). Another study from Australia identified that the presence of suicidal ideation was negatively associated with help‐seeking behavior (Calear et al., 2014).

### 4.2 Implications of study findings

The major implications of this study are threefold, First, it provides preliminary evidence of the reliability and validity of the LOSS and SOSS scales in a different religious and cultural setting. This demonstrates the applicability of these instruments to diverse populations and contexts. Next, it has identified setting specific knowledge gaps in suicide literacy that may be used to inform suicide awareness programs. Finally, data from the study will assist in developing strategies for dealing with stigmatizing attitudes to commit suicide and enhance suicide‐related awareness of society. We suggest that the validity of these scales be examined in the community‐related samples with different demographic attributes as suicide‐related stigma may vary as a function of age and educational attainment (Griffiths et al., 2008).

### 4.3 What is already known

Suicide is a neglected public mental health problem in Bangladesh where no attempt to determine the suicide literacy and stigma toward suicide was documented.

### 4.4 What this study adds

This study validated the two vital instruments LOSS‐B and SOSS‐B into Bangla those could be utilized in several settings in Bangladesh. It also revealed the level of suicide literacy and stigma among the university students of the country.

### 4.5 Future directions

Appropriate strategies should be designed to improve the literacy of suicide and reduce the stigma in Bangladesh. Future community‐based studies assessing the relationship of help‐seeking and level of literacy and stigma should be aimed.

### 4.6 Strengths and limitations

This is the first attempt to test the psychometric properties of the LOSS‐B and SOSS‐B as well as to determine the level of suicide literacy and stigma in Bangladesh. However, the study has several limitations. First, only internal consistency form of reliability was assessed without determining other forms such as test, retest, and inter‐rater. Second, we did not assess the detailed psychometric properties of the LOSS‐B as it is an edumetric instead of psychometric tool where there are correct answers rather than opinions/attitudes/behaviors. Third, data were collected from the university students that may restrict the generalization of study results. Fourth, samples were collected conveniently that might be a source of selection and response bias that hinders the generalization of study results.

## 5 CONCLUSION

This study culturally adapted the literacy of suicide scales and stigma of suicide scales into Bangla and determined the psychometric properties of the latter among university students in Bangladesh. The SOSS Bangla contains 13 items in three subscales (stigma five, isolation four, and glorification four). The instrument showed acceptable psychometric properties in Bangla. The suicide literacy was significantly higher in females, students of medicine, having a family history of suicidal attempts and a history of student nonfatal attempts while stigma was also significantly lower among the females and a history of past attempts. These instruments could be utilized in further research and academic and clinical settings.

## CONFLICT OF INTEREST

The authors declare no conflict of interest.

## AUTHOR CONTRIBUTIONS

*Conception and design*: S. M. Yasir Arafat. *Methods and instrument development*: S. M. Yasir Arafat, Fahad Hussain, and Md. Aminul Islam. *Data collection*: S. M. Yasir Arafat, Fahad Hussain, and Md. Aminul Islam. *Data analysis*: S. M. Yasir Arafat. *Drafting the manuscript*: S. M. Yasir Arafat and Vikas Menon. *Revision and approval*: All authors.

### PEER REVIEW

The peer review history for this article is available at https://publons.com/publon/10.1002/brb3.2432.

## ORCID

S. M. Yasir Arafat https://orcid.org/0000‐0003‐0521‐5708

## Data Availability

The data that support the findings of this study are available on request from the corresponding author.
